# Human milk and mucosa-associated disaccharides impact on cultured infant fecal microbiota

**DOI:** 10.1038/s41598-020-68718-4

**Published:** 2020-07-16

**Authors:** Antonio Rubio-del-Campo, Cristina Alcántara, María Carmen Collado, Jesús Rodríguez-Díaz, María J. Yebra

**Affiliations:** 1 Laboratorio de Bacterias Lácticas Y Probióticos, Departamento de Biotecnología de Alimentos, IATA-CSIC, Valencia, Spain; 2 Departamento de Microbiología, Facultad de Medicina, Universidad de Valencia, Valencia, Spain

**Keywords:** Microbiology, Molecular biology

## Abstract

Human milk oligosaccharides (HMOs) are a mixture of structurally diverse carbohydrates that contribute to shape a healthy gut microbiota composition. The great diversity of the HMOs structures does not allow the attribution of specific prebiotic characteristics to single milk oligosaccharides. We analyze here the utilization of four disaccharides, lacto-*N*-biose (LNB), galacto-*N*-biose (GNB), fucosyl-α1,3-GlcNAc (3FN) and fucosyl-α1,6-GlcNAc (6FN), that form part of HMOs and glycoprotein structures, by the infant fecal microbiota. LNB significantly increased the total levels of bifidobacteria and the species *Bifidobacterium breve* and *Bifidobacterium bifidum*. The *Lactobacillus* genus levels were increased by 3FN fermentation and *B. breve* by GNB and 3FN. There was a significant reduction of *Blautia coccoides* group with LNB and 3FN. In addition, 6FN significantly reduced the levels of *Enterobacteriaceae* family members. Significantly higher concentrations of lactate, formate and acetate were produced in cultures containing either LNB or GNB in comparison with control cultures. Additionally, after fermentation of the oligosaccharides by the fecal microbiota, several *Bifidobacterium* strains were isolated and identified. The results presented here indicated that each, LNB, GNB and 3FN disaccharide, might have a specific beneficial effect in the infant gut microbiota and they are potential prebiotics for application in infant foods.

## Introduction

Studies using in vitro analysis have shown that human milk unconjugated oligosaccharides (HMOs) and the glycan moiety of glycoproteins are effective in selectively promoting the growth of beneficial bacteria^1–3^. *Bifidobacterium longum, Bifidobacterium bifidum*, *Bifidobacterium breve* and *Bifidobacterium catenulatum* group are frequently isolated from breast-fed infant feces^4,5^. Some species of *Lactobacillus*, including *Lactobacillus reuteri*, *Lactobacillus casei* and *Lactobacillus rhamnosus* are also constituents of the infant gastrointestinal tract^6,7^. Among the bifidobacteria isolated from infant feces, there are important differences in the fermentation patterns of HMOs, which have more than two hundred distinct structures^8–10^, and this opens the possibility to accurately modulate the gut microbiota with specific HMOs^11^. To date only a few analyses have determined the physiological impact of individual HMOs on the infant gut microbiota. Thus, an in vitro fermentation system with stool samples from formula-fed babies demonstrated that lacto-*N*-biose (LNB), the main building block of type-1 HMOs, increased the total bifidobacterial population^12^. Especially *B. bifidum* numbers were highly induced in comparison to lactulose, raffinose, GOS or mannanoligosaccharides^12^. Lacto-*N*-neotetraose and 2′-fucosyllactose (2′FL) were fermented at a higher rate than 6′-sialyllactose in in vitro mixed culture systems by microbiota from breast or formula fed infants, but none of them showed an important effect in the number of bifidobacteria^13^. Total HMOs purified from human milk increased the number of *Bifidobacterium* spp. while the number of *Escherichia* spp. and *Clostridium perfringens* diminished during in vitro fermentation of infant microbiota^1^. In these cultures, most of the 2′FL, 3-fucosyllactose (3FL) and lactodifucotetraose (LDFT) from the HMOs supplement were consumed, suggesting that some specific oligosaccharides may be responsible of the prebiotic effect attributed to HMOs. A few studies using in vitro colon models^14^ and in vivo animal models also underline the ability of individual HMOs to modify the composition of the gastrointestinal microbiota^15,16^.

HMOs are present at high concentrations in human milk but not in infant formulas^17^. We have previously synthesized fucosyl- α-1,3-*N*-acetylglucosamine (3FN) and fucosyl-α-1,6-*N*-acetylglucosamine (6FN) using the transglycosylation activity of α-L-fucosidases^18^. 3FN forms part of HMOs^2^ and 6FN of *N*-glycosylated proteins, including mucins^19^. We have also produced LNB and galacto-*N*-biose (GNB), the core type-1 sugar from mucins, using the transgalactosylation activity of the β-galactosidase GnbG^20^. The synthesized fucosyl-*N*-GlcNAc disaccharides are fermented in vitro by pure cultures of *L. casei*, *L. rhamnosus*, *B. breve* and *B. pseudocatenulatum* species^21^. Regarding LNB, it was fermented in vitro for all the strains tested of *B. bifidum*, *B. infantis, B. longum* subsp. *longum* and *B. breve*, and for some strains of *B. pseudocatenulatum*, *Bifidobacterium animalis* subsp. *animalis* and *Bifidobacterium pseudolongum*^22^. In addition, we have seen that LNB and GNB are also fermented by pure cultures of *Lactobacillus* species^20^. In this work, we have investigated, in batch cultures using total microbiota isolated from stool samples of breastfed infants, the effects of LNB, GNB, 3FN and 6FN on infant microbiota composition and subsequent short chain fatty acids production. Our final aim is to compare the potential prebiotic effect of those four disaccharides.

## Results

### Total and specific bacterial levels modulation with human milk disaccharides

**Figure 1:**
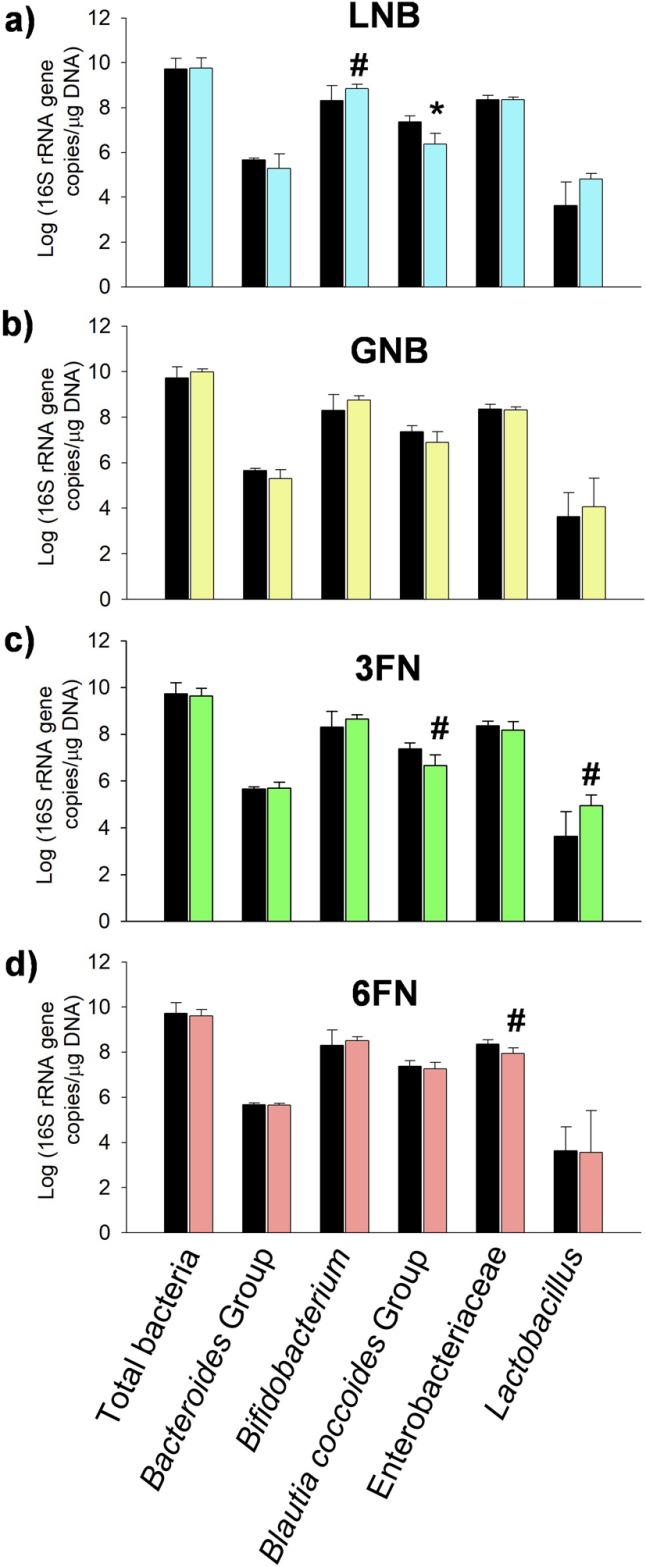
Total and specific bacterial levels measured with qPCR in cultures with fecal infant microbiota supplemented with oligosaccharides (clear bars). (**a**) lacto-*N*-biose (LNB); (**b**) galacto-*N*-biose (GNB); (**c**) fucosyl-α-1,3-*N*-acetylglucosamine (3FN); (**d**) fucosyl-α-1,6-*N*-acetylglucosamine (6FN). Control cultures are without carbohydrate added (dark bars). In all graphs, the data of the control is represented for better comparison. Data presented are mean values based on at least three replicates. Error bars indicate standard deviations. Statistically significant differences compared to control are indicated: ^#^*p* < 0.1; *p < 0.05.

The disaccharides LNB, GNB, 3FN and 6FN were synthesized by transglycosylation reactions as previously described^20,21^, and they were tested independently in fermentation assays, using total microbiota isolated from stool samples of breastfed infants. The addition of any of those oligosaccharides decreased the culture pH (Table 1). However, while the cultures with LNB and GNB resulted in a significant pH reduction (more than 2.5 pH units), the cultures with 3FN and 6FN showed a change in pH (about 1.0 pH unit) similar to the control culture without added sugar. These results are in agreement with the amount of oligosaccharides metabolized by the cultured infant microbiota, being the percentages of LNB and GNB consumed higher than the ones of the fucosylated oligosaccharides consumed (Table 1). Microbiota modulation was first determined at group and genus level by analyzing the numbers of total bacteria, *Bacteroides*, *Bifidobacterium*, *Blautia cocoides* group, *Enterobacteriaceae* family and *Lactobacillus* spp. Total bacterial numbers were similar among all the cultures, including the control (Fig. 1). Since the disaccharides were not completely consumed, that result suggested that they can be utilized only by a few selected groups of bacteria present in the infant microbiota. The results presented here (*p* = 0.0964, Dunnett’s test) (Fig. 1a) and previous studies, using fecal bacteria from formula-fed infants, showed that the total bifidobacterial community incremented with LNB^12^. Contrary to *Bifidobacterium* genus, a significant (*p* = 0.0192, Dunnett’s test) decrease in the number of cells during fermentation of LNB was observed for *Blautia coccoides* group (Fig. 1a). Regarding the fucosyl-*N*-acetylglucosamine disaccharides, the *Lactobacillus* genus is significantly (*p* = 0.0540, Dunnett’s test) increased by 3FN (Fig. 1c). 6FN has no significant effect in the cell numbers of lactobacilli respect to the control (Fig. 1d). Significantly (*p* = 0.0822, Dunnett’s test) lower cell numbers were observed for *Blautia coccoides* group in cultures containing 3FN and for *Enterobacteriaceae* family (*p* = 0.0751, Dunnett’s test) in cultures with 6FN (Fig. 1c and d).

**Table 1:** Decrease of pH values of culture supernatants and % of consumed oligosaccharides (mean ± standard deviation) from infant fecal microbiota fermentation assays.

	Control	LNB	GNB	3FN	6FN
pH decrease^a^	0.83 ± 0.32	2.97 ± 0.26*	2.89 ± 0.27*	0.98 ± 0.34	0.95 ± 0.33
% consumed disaccharides	-	82.33 ± 15.94	78.33 ± 19.40	41.92 ± 9.49	23.51 ± 15.57

Control, without sugar added; LNB, lacto-*N*-biose; GNB, galacto-*N*-biose; 3FN, fucosyl-α-1,3-*N*-acetylglucosamine; 6FN, fucosyl-α-1,6-*N*-acetylglucosamine. ^a^Statistical significant differences compared to control are indicated: *p < 0.05.

### *Bifidobacterium* and *Lactobacillus* species modulation with human milk disaccharides

**Figure 2:**
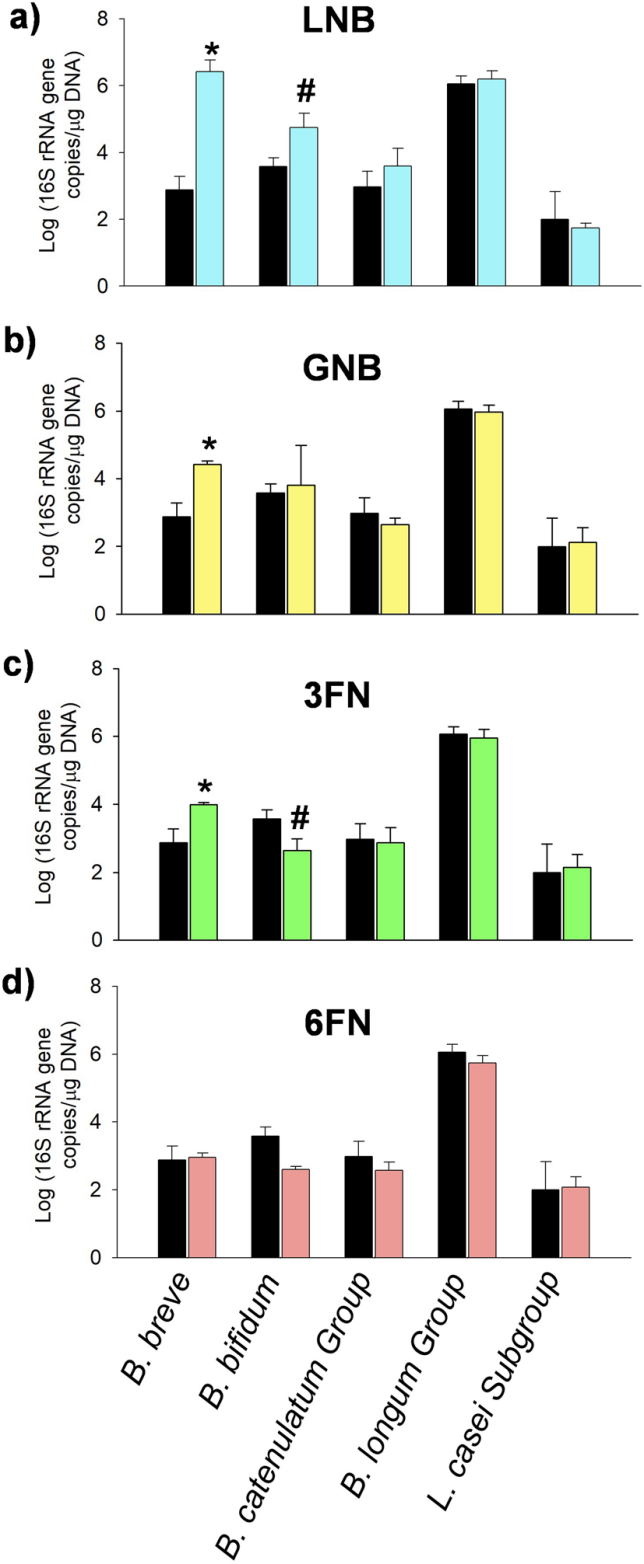
*Bifidobacterium* species/groups and *Lactobacillus casei* group levels measured with qPCR in cultures with fecal infant microbiota supplemented with oligosaccharides (clear bars). (**a**) lacto-*N*-biose (LNB); (**b**) galacto-*N*-biose (GNB); (**c**) fucosyl-α-1,3-*N*-acetylglucosamine (3FN); (**d**) fucosyl-α-1,6-*N*-acetylglucosamine (6FN). Control cultures are without carbohydrate added (dark bars). In all graphs, the data of the control is represented for better comparison. Data presented are mean values based on at least three replicates. Error bars indicate standard deviations. Statistically significant differences compared to control are indicated: ^#^*p* < 0.1; *p < 0.05.

In addition to *Bifidobacterium* and *Lactobacillus* abundance at the genus level, *Bifidobacterium* species (*Bifidobacterium breve*, *Bifidobacterium bifidum*, *Bifidobacterium catenulatum* group, *Bifidobacterium animalis* subsp. *lactis* and *Bifidobacterium longum* group) and *Lactobacillus* species (*Lactobacillus acidophilus, L. casei* subgroup and *Lactobacillus reuteri* subgroup) were also analyzed (Fig. 2). *B. lactis*, *L. reuteri* subgroup and *L. acidophilus* were below detection limit levels. LNB (*p* < 0.0001, Dunnett’s test), GNB (*p* < 0.0001, Dunnett’s test) or 3FN (*p* = 0.0011, Dunnett’s test) supplementation significantly increased the number of cells of *B. breve* compared to control culture (Fig. 2). In addition, *B. bifidum* was also incremented (*p* = 0.0808, Dunnett’s test) with LNB (Fig. 2a). In agreement with this, LNB has been previously shown as a substrate supporting the growth of strains belonging to *B. breve* and *B. bifidum*^22^. Although LNB is also a substrate for *B. longum* species^22^ and they were in a high proportion compared with the other analyzed strains (Fig. 2a), there was not a significant cell number increase in the presence of that carbohydrate. In this respect, previous results showed that bifidobacteria increment in cultures with prebiotics is higher when these bacteria are at lower levels^23^. The 16S rRNA gene copy numbers of total bifidobacteria are higher than the sum of the numbers of the four species (*B. breve*, *B. bifidum*, *B. catenulatum* group and *B. longum* group) tested here (Figs. 1 and 2). Although these species are usually the dominant bifidobacteria detected in the infant feces, the literature showed that there is an extraordinary variation in the individual composition of *Bifidobacterium* species in the infant gut^4^. Therefore, the discrepancies observed between the 16S rRNA gene copy numbers might be due to the presence of other *Bifidobacterium* species that outcompete those generally infant gut-associated bifidobacterial species. Regarding 3FN, previous studies demonstrated that *B. breve* strain ATCC15700 was able to metabolize it^21^. The numbers of *B. bifidum* declined significantly (*p* = 0.0927, Dunnett’s test) in cultures with 3FN and they showed a tendency to decrease with 6FN (Fig. 2c,d). Two extracellular cell-anchored α-L-fucosidases, AfcA and AfcB, that hydrolyze α-1,2- and α-1,3/1,4-linked fucosyl residues, respectively, have been characterized in that species^24,25^. However, neither 3FN nor 6FN were catabolized by *B. bifidum*^21^, which is in agreement with the fact that those enzymes do not hydrolyze them^26^. Interestingly no differences were observed in the numbers of *L. casei* subgroup (Fig. 2) although as mentioned above these species contain the metabolic pathways to utilize LNB, GNB and 3FN as energy sources. Therefore, these results suggested a competition with other bacterial species with probably higher affinity for those substrates.

### Lactate, formate and SCFA production changes with human milk disaccharides

Fermentation of non-digestible carbohydrates by the gut microbiota in the large intestine results in the accumulation of SCFA. About 90–95% of the SCFA present in the colon are acetate, propionate and butyrate^27^. Although lactate and formate are not SCFA, they are also major organic acids produced from the fermentation of carbohydrates by lactic acid bacteria^28,29^. Therefore, the concentration of lactate, formate and SCFA due to oligosaccharide fermentation by the infant fecal microbiota batch cultures were analyzed (Table 2). Lactate, formate and acetate increased significantly in the presence of LNB or GNB compared to the un-supplemented media control. Previous results with cultures containing LNB as carbon source and inoculated with fecal samples from formula-fed infants also showed an important acetate increment^12^. None of the tested sugars showed a butyrogenic effect in the cultures. Indeed, butyrate concentration significantly decreased with LNB and GNB fermentation (Table 2). Previous results demonstrated that oral administration of *B. breve* resulted in a decrease of fecal butyrate production in low birth weight infants^30^. As described above in the cultures supplemented with LNB or GNB significantly increased the cell counts of that species (Fig. 2), which might have a role lowering butyric acid levels. Additionally, *Bacteroides* and *Blautia coccoides* groups contain butyric acid producing genera^31^ that likely derive amino acids present in the culture media towards the synthesis of butyrate^32^. The availability of LNB or GNB in the cultures resulted in a tendency to decrease those bacterial groups with respect to the control (Fig. 1) and this could result in less production of butyric acid.

**Table 2:** Lactate, formate and short-chain fatty acids concentration (mM) (mean ± standard deviation) in infant fecal microbiota fermentation with oligosaccharides.

	Control	LNB	GNB	3FN	6FN
Lactate	1.12 ± 0.14	4.93 ± 1.73*	4.48 ± 3.71*	1.06 ± 0.154	0.78 ± 0.36
Formate	2.43 ± 0.67	3.69 ± 0.38*	4.62 ± 0.44*	2.64 ± 0.91	2.77 ± 0.85
Acetate	3.67 ± 0.83	9.19 ± 5.44*	7.55 ± 1.78^#^	3.67 ± 1.07	3.61 ± 1.12
Propionate	1.45 ± 0.94	1.80 ± 0.86	3.07 ± 2.02	1.15 ± 0.62	0.87 ± 0.58
Butyrate	11.96 ± 3.80	6.84 ± 2.69^#^	6.75 ± 4.85^#^	10.62 ± 2.47	11.23 ± 1.61

Control, without sugar added; LNB, lacto-*N*-biose; GNB, galacto-*N*-biose; 3FN, fucosyl-α-1,3-*N*-acetylglucosamine; 6FN, fucosyl-α-1,6-*N*-acetylglucosamine. Statistical significant differences compared to control are indicated: ^#^*p* < 0.1; *p < 0.05.

### Identification of isolated *Bifidobacterium* species

**Figure 3:**
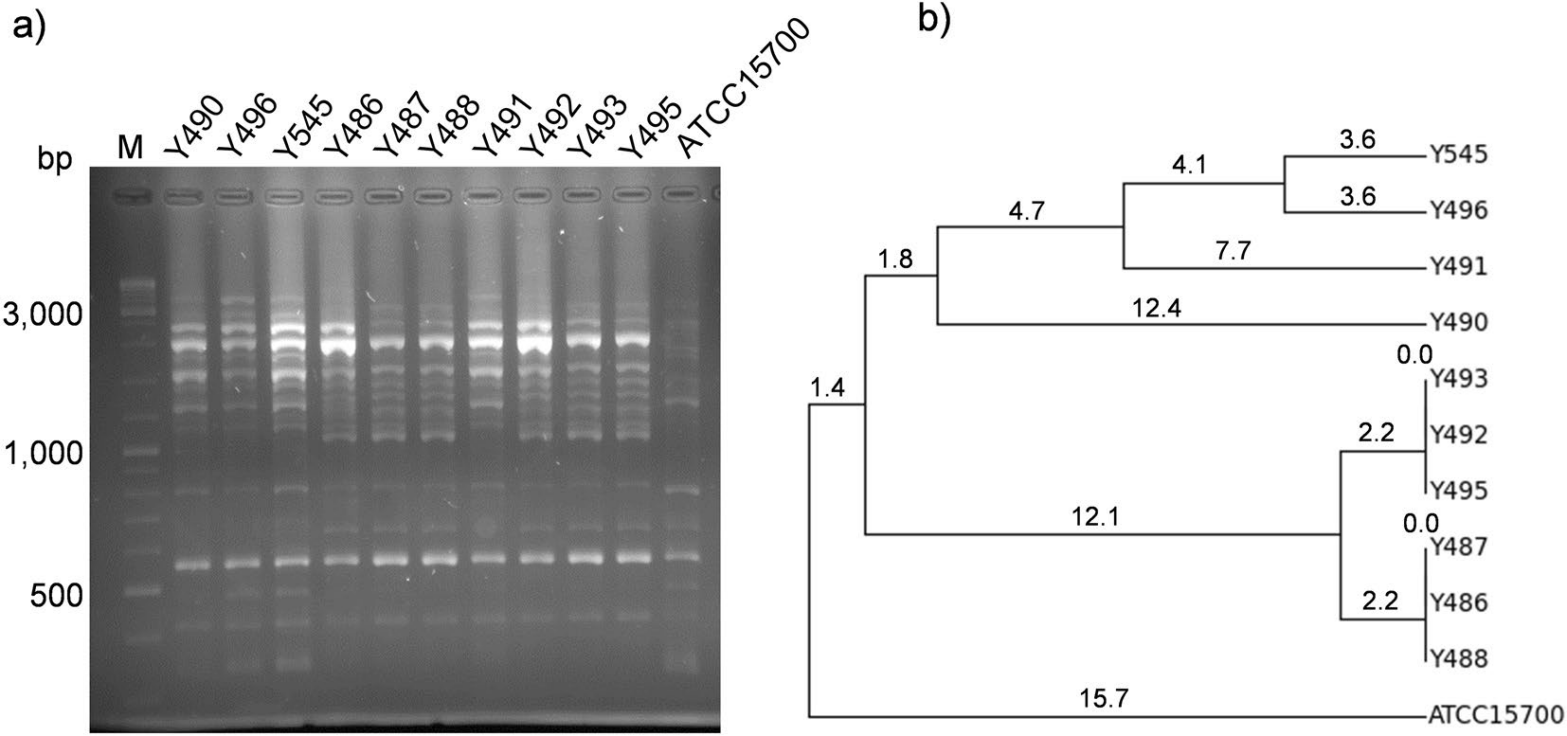
Evaluation of the genetic diversity among the *Bifidobacterium breve* strains isolated in this study using RAPD-PCR analysis. Agarose gel (2%) electrophoresis (**a**) and cluster analysis (**b**) of the DNA band patterns obtained from the *B. breve* strains Y486, Y487, Y488, Y490, Y491, Y492, Y493, Y495, Y496 and Y545. The type strain *B. breve* ATCC15700 was used as control. Cluster analysis was performed using the unweight pair group method with arithmetic mean (UPGMA) analysis. The numbers in the dendrogram provide a measure of genetic distances between isolates. Lane M, DNA molecular weight marker.

**Figure 4:**
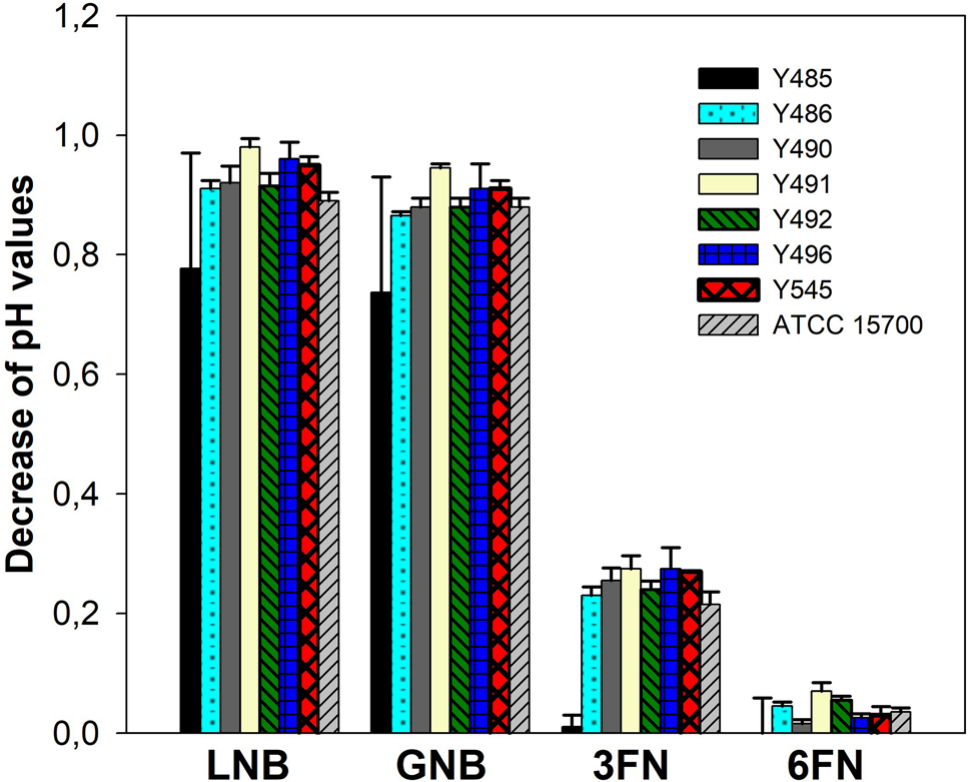
Decrease of pH values of culture supernatants from *Bifidobacterium longum* strain Y485 and *Bifidobacterium breve* strains Y486, Y490, Y491, Y492, Y496, Y545 and ATCC 15,700 cultured in MRS basal medium supplemented with 10 mM lacto-*N*-biose (LNB), galacto-*N*-biose (GNB), fucosyl- α-1,3-*N*-acetylglucosamine (3FN) or fucosyl-α-1,6-*N*-acetylglucosamine (6FN). Data presented are mean values based on at least three replicates. Error bars indicate standard deviations.

Samples from the disaccharide fermentation cultures were plated on MRS with mupirocin and cysteine in order to isolate *Bifidobacterium* species. Isolates were subjected to RAPD-PCR analysis and at least one representative of each band pattern was kept for subsequently species identification. Partial 16S rRNA gene sequencing led to the identification of one isolate as *B. longum* (Y485) and ten isolates as *B. breve* (Y486, Y487, Y488, Y490, Y491, Y492, Y493, Y495, Y496 and Y545). This result is in line with the fact that the latter species was stimulated by three out of the four disaccharides tested (Fig. 2). However, there are a number of cells of *B. longum* present in a high proportion in the cultures and therefore, other explanations for that result cannot be discarded^33^. Previous studies proved that RAPD-PCR analysis is an appropriate molecular tool to differentiate bifidobacteria at the strain level^34^. The RAPD-PCR profiles were almost similar in all isolated *B. breve* strains, suggesting that they were closely related (Fig. 3). However, the cluster analysis of RAPD-PCR profiles allowed differentiating the ten isolates to at least six different strains (Fig. 3). The *B. longum* strain Y485 and the *B. breve* strains Y486, Y490, Y491, Y492, Y496 and Y545 were selected to characterize their ability to utilize LNB, GNB, 3FN and 6FN. The *B. breve* type strain (ATCC 15700) was also included in this analysis. The ability of this strain to ferment 3FN was already demonstrated in our lab and it was used here as a positive control^21^. The results demonstrate that all the *Bifidobacterium* strains tested were able to ferment LNB and GNB as evidenced by the decreased pH of the culture media (Fig. 4). 3FN was not utilized by *B. longum* strain Y485 but it was fermented by all the *B. breve* strains tested. None of the strains fermented 6FN (Fig. 4).

## Discussion

Studies using in vitro analysis and animal models suggested that human milk oligosaccharides (HMOs) are crucial for modulating the infant gut microbiota and exerting important health benefits, including prevention of pathogen attachment and immunomodulation^2,35–37^. However, HMOs are not present or are present in low concentrations in infant formulas^17^. Therefore, there is a need to evaluate the influence of individual carbohydrates in the microbiota profile of newborns for their selection as prebiotic substrates. In this work, the disaccharides LNB, GNB, 3FN and 6FN, that form part of HMOs and mucosa-associated glycans, were tested individually in fermentation assays, using total microbiota isolated from stool samples of breastfed infants. Total levels of bifidobacteria were significantly increased by LNB. The effect of this disaccharide was previously analyzed in vitro on fecal bacteria from formula-fed infants and the total bifidobacterial community was also incremented^12^. These findings and our results confirmed LNB as an important growth-promoting factor for the genus *Bifidobacterium*. The transport and catabolism of LNB as well as GNB are mediated by the proteins encoded by the LNB/GNB gene cluster present in several *Bifidobacterium* species^38–41^. This gene cluster includes a key enzyme, the LNB/GNB phosphorylase (LNBP), which catalyzes the cleavage of the 1,3-β-glycosidic bond of LNB or GNB into galactose-1-phosphate and GlcNAc or GalNAc, respectively^42^. LNBP from *Bifidobacterium* species showed similar affinity for LNB and GNB, however, phosphorylases specific to GNB has also been characterized in intestinal bacteria^43^, which might explain the significant and nonsignificant differences found here between LNB and GNB, respectively, on *Bifidobacterium* cell counts (Fig. 1). Regarding lactobacilli, the operon *gnb* involved in the metabolism of LNB and GNB has been characterized in *L. casei*^44^. In this bacterium, those disaccharides are transported and phosphorylated by the PTS^Gnb^ and once inside the cell are hydrolyzed by the specific phospho-β-1,3-galactosidase GnbG. In addition to *L. casei*, the species *Lactobacillus rhamnosus*, *Lactobacillus zeae*, *Lactobacillus gasseri* and *Lactobacillus johnsonii* are also able to utilize LNB and GNB as a carbon source^20^. However, while *L. rhamnosus* and *L. zeae* contain a phospho-β-galactosidase homologous to GnbG, *L gasseri* and *L. johnsonii* do not have GnbG homologs, suggesting that the catabolism of both sugars in the latest species must depend on other glycosidases^20^.

Our results showed that the cell numbers of the *Bacteroides* and *Blautia coccoides* groups have a tendency to decrease during fermentation of LNB or GNB. BLAST searches using the amino acid sequence of LNBP from *B. bifidum*^22^ against the phylum *Bacteroidetes* did not show relevant homology with any protein. Analysis using GnbG from *L. casei*^44^ showed significant homology with proteins from several species belonging to *Bacteroidetes*, such as *Sphingobacterium faecium*^45^, *Hymenobacter* sp.^46^, *Chryseobacterium* sp.^47^ or *Mucilaginibacter* sp.^48^. However, the primers used to target *Bacteroides* group^49^ do not amplify the 16S rRNA gene of those genera and in addition, they are isolated from environments different from the intestine. These results suggested the absence of enzymes homologs to LNBP and GnbG in gut species of the *Bacteroides* group, however, other enzymatic strategies to metabolize those disaccharides cannot be discarded. Alternatively, the decrease of *Bacteroides* group counts might also be explained by the pH reduction determined in cultures with LNB or GNB since some *Bacteroides* species showed a poor growth at low pH^50^. Regarding *B. coccoides* group, some species contain homologs to LNBP and GnbG, although the possible role of these enzymes on LNB and GNB metabolism needs to be investigated.

The fucosyl-disaccharide 3FN is hydrolyzed by the α-L-fucosidase AlfB from *L. casei*^51^ and the ability of this bacterium to ferment 3FN rely upon this enzyme^21,52^. Homologs to AlfB are present in strains of the *L. casei*/*L. paracasei*/*L. rhanmosus* group and other lactobacilli species, including *Lactobacillus sharpeae*, *Lactobacillus fabifermentans*, *Lactobacillus paralimentarius*, *Lactobacillus tucceti*, *Lactobacillus ghanensis*, *Lactobacillus apis*, *Lactobacillus vini*, *Lactobacillus gigeriorum*, *Lactobacillus panisapium* and *Lactobacillus helsingborgensis* (https://www.ncbi.nih.gov/genome). In accordance with this, the *Lactobacillus* genus is significantly increased by 3FN (Fig. 1c), although whether the lactobacilli present in the fermentation cultures analyzed here contain homologs to AlfB would require further analysis. As well, other catabolic systems different to those already characterized for 3FN utilization might exist. Regarding 6FN, lactobacilli cell counts were not affected. Curiously, 6FN is cleaved by the α-L-fucosidase AlfC from *L. casei* strain BL23^51^, but unlike 3FN, it is not a metabolic substrate for this bacterium. The *alfC* gene forms part of the *alf2* operon that is induced by the glycoamino acid 6FN-Asn, a constituent of the mammalian *N*-glycoproteins, but not by its glycan moiety 6FN^53^. *L. casei* strain BL87 and *L. rhamnosus* BL327 contain homologs to AlfC and they are able to metabolize 6FN although with low efficiency^21^. BLAST searches using the amino acid sequences of AlfB and AlfC from *L. casei*^51^ against *Blautia coccoides* group and *Enterobacteriaceae*, respectively, did not show relevant homology with any protein, suggesting the absence of enzymes homologs to those α-L-fucosidases. However, the presence on those bacterial groups of other enzymes able to catabolize 3FN and 6FN cannot be ruled out. The reduced numbers of *Enterobacteriaceae* in the presence of 6FN, might be relevant for specific infant gastrointestinal disorders such as the development of necrotizing enterocolitis, which has been associated with an increased relative abundance of *Enterobacteriaceae* strains^54^.

The major products of non-digestible carbohydrate metabolism by the gut microbiota are SCFA and organic acids such as formate and lactate. Both, organic acids and SCFA, resulted in a decrease of the luminal pH, which in turn resulted in inhibition of pathogens growth and increment of nutrients absorption^55^. SCFA are a source of energy for colonocytes^56^ and also for some members of the microbiota^31^. In addition, SCFA have important roles in colorectal cancer, immune system and inflammatory responses^57^. The fermentation of LNB and GNB by the infant microbiota resulted in an increment of the production of lactate, formate and acetate (Table 2). According to this, *Bifidobacterium* species produced both acetate and lactate through the “bifid-shunt” route during carbohydrate fermentation^58^. Stool pH of breastfed infants is lower than that of bottle-fed infants^59^ and it is due to the high lactate and acetate levels present in the feces of breastfed infants^11,60^. Interestingly, research using an animal model has demonstrated that increased production of acetate by bifidobacteria protects the host against enteropathogenic infection^61^. Lactate has also been shown to be important in maintaining intestinal barrier function through stimulation of enterocyte proliferation in a murine model^62^. Although the cell numbers of lactobacilli and *B. breve* increase after fermentation of 3FN by the infant microbiota, there is not an increment in the production of lactate. Previous studies have shown that in anaerobiosis the L-fucose metabolism by some bacteria produces more efficiently 1,2-propanediol than lactate^63,64^. However, this metabolite has not been detected in the supernatants from the 3FN-supplemented cultures (data not shown), suggesting that either it was not produced or if produced, it was further metabolized by other members of the microbiota present in the fermentation cultures. Some bacteria utilize 1,2-propanediol, via propionaldehyde, to produce propionate and/or propanol^31^. Since the concentration of propionate did not increase with 3FN, the possible 1,2-propanediol produced would be derived to propanol. Whether the lactobacilli and bifidobacteria present in the fermentation cultures analyzed here produce 1,2-propanediol, that is further derived to propanol by other bacteria also present in the cultures, is unknown. Additional studies will be needed to find out why the lactate levels do not change in the cultures with 3FN.

This study demonstrates that the human milk and mucosa-associated disaccharides LNB, GNB and 3FN and 6FN are fermented in vitro by the microbiota isolated from stool samples of breastfed infants. The results presented here allow a comparison among four individual disaccharides and they indicated that each disaccharide distinctly modulates the microbiota composition and activity. The growth of specific *Bifidobacterium* species is significantly promoted by LNB, GNB and 3FN, and the growth of lactobacilli by 3FN. Furthermore, LNB and GNB enhance the production of lactate, formate and acetate. Regarding 6FN, *Enterobacteriaceae* family counts are significantly reduced, although further work is needed to determine the relevance of this finding. Overall, our results highlight the prebiotic potential of each LNB, GNB and 3FN for application in functional infant foods.

## Methods

### Enzymatic synthesis and purification of human milk disaccharides

The disaccharides LNB, GNB, 3FN and 6FN were produced in our laboratory as previously described^18,20,21^. Briefly, transgalactosylation reactions containing 100 mM Tris–HCl buffer, pH 7.5, *o*-nitrophenyl β-D-galactopyranoside 40 mM, 0.17 U/ml of GnbG and *N*-acetylglucosamine (GlcNAc) 200 mM or *N*-acetylgalactosamine (GalNAc) 200 mM were carried out at 42 °C for 3 h to synthesize LNB and GNB, respectively. Transfucosylation reactions containing 100 mM Tris–HCl buffer, pH 7.0, *p*-nitrophenyl α-L-fucopyranoside 50 mM, *N*-acetylglucosamine (GlcNAc) 150 mM and 180 U/ml of AlfB or 800 U/ml AlfC, were carried out at 42 °C for 15 min (AlfB) and 10 min (AlfC) to synthesize 3FN and 6FN, respectively.

The transglycosylation reaction products were purified by high-performance liquid chromatography (HPLC) with a Jasco PU2080Plus system coupled to a refractive index detector (Jasco RI-2031 Plus) using a preparative Rezex RCM-Monosaccharide column (Phenomenex, Torrance, CA, USA), as described previously^21^. Based on HPLC analysis, the purity of the synthesized oligosaccharides was found to be more than 99%.

### Batch culture fermentation assays

Stool samples from five healthy infants exclusively breast-fed and two months old were selected from a previous study^65^. A pooled fecal sample was used to isolate the microbiota as previously described^66^. Two grams of feces were homogenized in 18 ml of physiological serum (NaCl 0.9%), placed on top of 3.5 ml Nycodenz 80% solution (Alere Technologies AS) and centrifuged for 1 h at 3,327 xg (Hermle Z383K). The layer containing the microbiota was collected and stored in 20% glycerol at – 80 °C. This microbiota was used to inoculate two ml of basal medium containing bactopeptone (Difco), 2 g/L; yeast extract (Pronadisa), 2 g/L; NaCl 0.1 g/L; K_2_HPO_4_ 0.04 g/L; KH_2_PO_4_ 0.04 g/L; MgSO_4_·7H_2_O 0.01 g/L; CaCl_2_·H_2_O 0.01 g/L; NaHCO_3_ 2 g/L; L-cysteine 0.5 g/L; bile salts 0.5 g/L; Tween 80 2 ml/L; haemin solution 0.05 g/L (Sigma); vitamin K_1_ (Sigma), 10 μl/L; and resazurin 0.025% solution, 4 ml/L. The pH of the medium was adjusted to 7.4. Four cultures were initiated with the purified disaccharides, LNB, GNB, 3FN or 6FN at 10 mM. A control culture without carbohydrate added was also included. Cultures were grown at 37 °C under anaerobic conditions using an anaerobic atmosphere generation system (Anaerogen, Oxoid), and at 48 h they were centrifuged, and pellets and supernatants were collected. Three independent cultures were carried out for each oligosaccharide.

### Quantification of bacterial groups, genera and species by specific real-time PCR

Bacterial DNA was extracted from each pellet of the cultures using the MasterPure DNA extraction Kit (Epicentre) according to the manufacturer’s instructions with some modifications. These include a previous treatment with lysozyme and mutanolysin for 60 min at 37 °C followed by cell disruption with 0.1 mm diameter glass beads. DNA concentration was measured using a Qubit 2.0 Fluorometer (Life Technology).

Quantitative real-time PCR (qPCR) assays were performed as previously described^67^ and using a series of bacterial group, genus and species-specific 16S rRNA gene primer pairs^49,67–74^ listed in supplementary Table S1. The qPCR amplification and detection were performed in a LightCycler 480 Real-Time PCR System (Roche Technologies). Each reaction mixture (10 μl) contained SYBR Green PCR Master Mix (Roche), 0.25 μl of each primer (10 μM) and 1 μl of template DNA. All samples were analyzed in duplicate. Standard curves obtained by using serial tenfold dilutions of specific DNA fragments were used to calculate bacterial concentration in each sample. A detection limit of 100 copies per reaction was established.

### Analysis of disaccharide consumption

To determine the carbohydrates present in the supernatants from the infant fecal microbiota fermentation assays, filtered culture supernatants were analyzed by high-pH anion-exchange chromatography with pulsed amperometric detection in a ICS3000 chromatographic system (Dionex) using a CarboPac PA100 column. A gradient of 10 to 100 mM NaOH was used at 27 °C for 15 min at a flow rate of 1 ml/min. Disaccharides were confirmed by comparison of their retention times with those of standards. The obtained calibration curves for LNB, GNB, 3FN and 6FN were linear in the range tested, 0.05–0.25 mM.

### Lactate, short-chain fatty acids and 1,2-propanediol analysis

Filtered culture supernatants were analyzed by HPLC with a Jasco PU2080Plus system coupled to a UV (210 nm) or refractive index (Jasco RI-2031Plus) detectors and using a Rezex ROA-Organic Acid or Rezex RCM-Monosaccharide columns (Phenomenex, Torrance, CA, USA). Lactic, formic, acetic, propionic, butyric acids (Rezex ROA-Organic Acid column) and 1,2-propanediol (Rezex RCM-Monosaccharide column) were determined as previously described^63,75^. The column temperatures were kept at 40 °C and 80 °C, respectively, in phosphoric acid 0.1% or water.

### Isolation of *Bifidobacterium* species

A dilution series was made from the LNB, GNB, 3FN and 6FN fermentation cultures, and 0.1 ml aliquots were plated on MRS agar with mupirocin 50 mg/L and cysteine 0.1%. These plates were incubated in anaerobic jars at 37 °C for 72 h in order to isolate *Bifidobacterium* species. More than ten colonies from each of the four disaccharide-supplemented cultures were selected until reaching a total number of fifty colonies. These were subsequently subjected to randomly amplified polymorphic DNA (RAPD) analysis. Each RAPD-PCR reaction (25 μl) contained bacterial cells from the colonies as source of DNA template, random primer MCV^76^ described in Table S1 at a final concentration of 2 μM, 400 μM of each dNTP, MgCl_2_ 4 mM, dimethyl sulfoxide (DMSO) 4%, 2 U of *Taq* DNA polymerase and PCR buffer (NZYtech). Amplifications were performed at 96 °C for 7 min, followed by 35 cycles of 30 s at 95 °C, 45 s at 30 °C and 1 min at 72 °C and extension for 7 min at 72 °C. Samples were analyzed by agarose gel electrophoresis and one representative isolate of each band pattern was kept for further analysis. Partial 16S rRNA gene of these bacterial isolates was amplified by PCR using cells from the colonies as the template and the primers 27F^77^ and 924R^78^ (Table S1). The PCR products were sequenced by the Central Service of Research Support of the University of Valencia (Spain). The sequences were used in BLAST searches to identify each isolated.

Genetic distance between the *B. breve* isolates was calculated using the Dice coefficient of similarity and the strains were clustered in a dendrogram using the unweight pair group method with arithmetic mean (UPGMA) analysis with PyElph 1.4 software tool^79^.

The growth of individual *B. longum* and *B. breve* strains in the presence of each disaccharide at 10 mM was tested in MRS basal medium using the culture conditions previously described for the *Bifidobacterium* strains^21^.

### Statistical analysis

One-way ANOVA with Dunnett’s multiple comparisons test was performed using GraphPad Prism, version 6.07 (GraphPad Software Inc., San Diego, CA, USA) and it was used to detect statistically significant differences between the control and the disaccharide-supplemented groups (LNB, GNB, 3FN and 6FN) on the following: numbers of total and specific bacterial groups, genera and species; pH decrease of culture supernatants; lactate, formate and SCFA. Statistical significance was accepted at two levels ^#^*p* < 0.1; *p < 0.05.

### Nucleotide sequence accession numbers

The partial nucleotide sequence of the 16S rRNA gene amplicons have been deposited at the GenBank database under the accession numbers MN650609 to MN650619.

### Ethical statement

All applicable international, national, and/or institutional guidelines for the use of human samples were followed. Samples were selected from the project “The power of maternal microbes on infant health (MAMI)”. The study protocol with the registration number 2015/0024 was approved by the Ethics Committee of Spanish National Research Council (CSIC). Written informed consent is obtained from a parent and/or legal guardian.

### Data availability statement

All data generated or analyzed during this study are included in this published article.

## Supplementary information

Supplementary file1 (PDF 293 kb)
