# Supplementary material for: Human milk and mucosa-associated disaccharides impact on cultured infant fecal microbiota
Source: Sci Rep. 2020 Jul 16;10:11845. doi: 10.1038/s41598-020-68718-4 (PMC7366668; doi:10.1038/s41598-020-68718-4)
Supplement: Supplementary file 1 — Supplementary file1 (PDF 293 kb) [file 41598_2020_68718_MOESM1_ESM.pdf]

Human milk and mucosa-associated disaccharides impact on cultured infant fecal microbiota

Antonio Rubio-del-Campo[1], Cristina Alcántara[1], María Carmen Collado[1], Jesús Rodríguez-Díaz[2;*] and María J. Yebra[1,*]

[1]Laboratorio de Bacterias Lácticas y Probióticos, Departamento de Biotecnología de Alimentos, IATA-CSIC, Valencia, Spain. [2]Departamento de Microbiología, Facultad de Medicina, Universidad de Valencia, Valencia, Spain.

Supplementary_Table S1. Primers used in this study

qPCR<br>Target bacteria	Name	Sequence	Reference
Total bacteria	pUniv1	CGT GCC AGC AGC CGC GG	Mira-Pascual et al., 2015
	pUniv2	TGG ACT ACC AGG GTA TCT	
Bacteroides group	Bac group F	GAG AGG AAG GTC CCC CAC	Echarri et al., 2011
	Bac group R	CGC KAC TTG GCT GGT TCA G	
Bifidobacterium genus	Bifido5'	GAT TCT GGC TCA GGA TGA	Gueimonde et al., 2004
	Bifido3'	CTG ATA GGA CGC GAC CCC	
Blautia coccoides group	g-Ccoc-F	AAA TGA CGG TAC CTG ACT AA	Matsuki et al., 2002
	g-Ccoc-R	CTT TGA GTT TCA TTC TTG	
Enterobacteriaceae family	EnterobactF	CATTGACGTTACCCGCAGAAGA	Bartosch et al., 2004
	EnterobactR	CTCTACGAGACTCAAGCTTGC	
Lactobacillus spp.	LacF	AGC AGT AGG GAA TCT TCC A	Mira-Pascual et al., 2015
	LacR	CAC CGC TAC ACA TGG AG	
Bifidobacterium breve	Bbreve5'	AAT GCC GGA TGC TCC ATC	Gueimonde et al., 2007
	Bbreve3'	GCC TTG CTC CCT AAC AAA	
Bifidobacterium bifidum	Bbifidum5'	TGA CCG ACC TGC CCC ATG CT	Gueimonde et al., 2007
	Bbifidum3'	CCC ATC CCA CGC CGA TAG	
Bifidobacterium catenulatum group	Bcatenulatum5'	GCC GGA TGC TCC GAC TCC T	Gueimonde et al., 2007
	Bcatenulatum3'	ACC CGA AGG CTT GCT CCC	
Bifidobacterium lactis	Anim-Lactis F	TCACGACAAGTGGGTTGCCA	Sheu et al., 2010
	Anim-Lactis R	GTTGATCGGCAGCTTGCCG	
Bifidobacterium longum group	Blongum5'	TTC CAG TTG ATC GCA TGG	Gueimonde et al., 2007
	Blongum3'	GGC TAC CCG TCG AAG CCA	
Lactobacillus acidophilus	LbAcF	CTGCTGTTTCTTCAGCATCT	Larsen et al., 2011
	LbAcR	TCAGTATTGATACCACGTGAAT	
Lactobacillus casei subgroup	sg_Lcas-F	ACC GCA TGG TTC TTG GC	Matsuda et al., 2009
	sg_Lcas-R	CCG ACA ACA GTT ACT CTG CC	
Lactobacillus reuteri subgroup	sg-Lreu-F	GAA CGC AYT GGC CCA A	Matsuda et al., 2009
	sg-Lreu-R	TCC ATT GTG GCC GAT CAG T	
	**Name**	**Sequence**	**Reference**
RAPD-PCR	MVC	AGT CAG CCA C	Tynkkynen et al., 1999
16S rRNA gene	27F	AGA GTT TGA TCC TGG CTC AG	Weisburg et al., 1991<br>Rudi et al., 1997
	924R	CTTGTGCGGGCCCCCGTCAATTC	
